# A Digital Stereomicroscopic Study of the Furcation Wall Thickness of Mesiobuccal Roots of Maxillary First and Second Molars

**Published:** 2010-05-20

**Authors:** Arash Shahravan, Alireza Rekabi, Hasan Shahabi, Rezvan Ashuri, Ali Mirzazadeh, Maryam Rad, Jahangir Haghani

**Affiliations:** 1. Department of Endodontics, Dental School, Kerman University of Medical Sciences, Kerman Oral and Dental Diseases Research Center, Kerman, Iran.; 2. Department of Endodontics, Dental School, Kerman University of Medical Sciences, Kerman, Iran.; 3. MPH in Epidemiology and Biostatistics, Physiology Research Center, Kerman University of Medical Sciences, Kerman, Iran.; 4. Department of Oral Medicine, Dental School, Kerman University of Medical Sciences, Kerman Oral and Dental Diseases Research Center, Kerman, Iran.; 5. Department of Radiology, Dental School, Kerman University of Medical Sciences, Kerman Oral and Dental Diseases Research Center, Kerman, Iran.

**Keywords:** Canal Thickness, Dentin, Furcal Wall, Maxilla, Molar, Thickness, Tooth Root

## Abstract

**INTRODUCTION:**

This study aimed to compare the thickness of the mesiobuccal furcal canal walls of first and second maxillary molars.

**MATERIALS AND METHODS:**

This study was performed on total of 30 first and second extracted molar teeth (15 each). The mesiobuccal roots of each tooth were separated at the cement-enamel junction (CEJ) level and embedded in acrylic resin. The embedded roots were cut horizontally at two and four mm below the CEJ using a 0.20 mm blade (overall three sections CEJ, two and four mm below). Next, photographs of all the horizontal sections were taken using a digital stereomicroscope with identical magnifications (×20). Using the photographs, two independent observers measured and recorded the minimal thickness from the canal wall of the first mesiobuccal (MB1) and the second mesiobuccal (MB2) canals to the furcation area. Data analysis was performed by repeating the measurement Analysis of Variance. The analysis was completed by making paired comparisons using the Bonferroni alpha adjustment method. Statistical significance was set at P<0.05.

**RESULTS:**

In maxillary first molars, the mean (±SD) thickness of the canal furcal wall MB1 in three sections were significantly higher than thickness of the canal furcal wall in MB2 (P<0.05). However there was no statistical difference between the mean (±SD) thickness of the canal furcal wall in the three sections (CEJ, 2 and 4 mm below) of maxillary second molars MB1 and MB2 canals.

**CONCLUSION:**

Only the maxillary first molars demonstrated significantly lower furcal canal wall thickness (FCWT) values in the MB2 canals. Maxillary second molars MB canals did not demonstrate statistical difference in FCWT values.

## INTRODUCTION

It is important to be familiar with tooth anatomy and its variations prior to any mechanical instrumentation (cleaning and shaping) of a root canal system [1][2]. Most maxillary molars are known to have a fourth canal (called second mesiobuccal or MB2) which is located palatal to the main or first mesiobuccal (MB1) canal; usually located in the mesiobuccal root [3]. Failure to find and treat the MB2 canal will lead to a poor long-term prognosis [4]. Usually, the preparation of MB2 canals in maxillary molars is a difficult procedure. It is recommended that the dental surgeon use operating microscopes during the preparation of MB2 canals [5][6][7].

In addition, the risk of strip perforation must be considered, especially in preparing the MB1 and MB2 canals of maxillary molars. For instance, Abou-Rass et al. demonstrated the risk of strip perforation in buccal roots of maxillary molars [8], and in another study, Prakash et al. indicated that MB2 canals were smaller and usually narrower than MB1 canals [9]. The authors have noted that in order to reduce the risk of furcal perforation, anti curvature filing has been used to remove more dentine from the mesial wall and less from the distal wall of the mesiobuccal canals [10]. Using this technique, filing is done while pressure is applied away from furcation wall [10]. The thickness of the MB canal on the furcation side is one of the most important factors that should be considered before canal instrumentation, due to preventing strip perforation in maxillary molars.

In practice, even with routine root canal preparation, the authors have reported cases with strip perforations in MB2 canals in maxillary molars. The size of the furcal canal wall thickness (FCWT) is a possible factor which encourages strip perforations. A review of literature did not provide any data or protocols for comparing furcal dentin thickness of the MB1 and MB2 canals of maxillary molars. The purpose of this study was to compare the FCWT of the MB1 and MB2 canals of the maxillary first and second molars at the cementoenamel junction (CEJ) level, as well as two and four millimeter below this junction.

## MATERIALS AND METHODS

Fifteen extracted maxillary first and fifteen maxillary second molar teeth, were selected after preliminary x-ray examinations and access preparations. The following criteria was used: teeth which have 1) three separated roots (Mesiobuccal, Distobuccal and Palatal) with separated orifices for MB1 and MB2; 2) no root fractures or cracks; 3) no visible external root resorption; 4) no evidence of previous root canal therapy; 5) no obvious root calcification, clinically and radiographically.

After scaling with an ultrasonic device, the teeth were submerged in NaOCl 5.25% for 30 minutes to eliminate remaining organic residues [11]. Subsequently the teeth were preserved in saline solution.

MB roots were separated from the CEJ. Afterwards, these roots were embedded in acrylic resin, using small cylindrical investment molds so that the CEJ was placed higher than the resin (exposed part). The long axis of each single root was oriented parallel to the mold wall. The apical foramen was also sealed with a small piece of red dental wax. In order to place the long axis of the MB roots in a parallel position with the acrylic cube walls, a size B spreader (Dentsply Maillefer, Ballaigues, Switzerland) was inserted into the MB1 canal of each root and then the handle of the spreader was oriented parallel to the walls of the acrylic cube with a Ney dental surveyor (Dentsply Prosthetic). After complete setting of the resin, the mold was released from the cube. A guiding groove was prepared along the surface of the resin block adjacent to the buccal side of the root.

**Figure 1:**
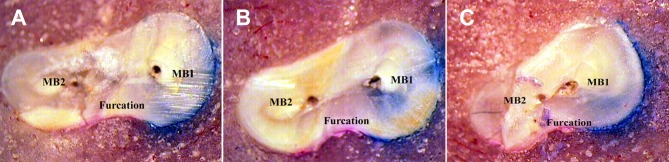
MB1 and MB1 canals of first Maxillary molar. A) at CEJ, B) 2 mm lower CEJ, C) 4 mm lower CEJ

Next, the embedded roots were cut horizontally in a sectioning machine at 2 and 4 mm below the CEJ with a 0.20 mm low speed saw, resulting in 45 sections for each type of maxillary molar. The coronal side of the section was then photographed with a digital stereomicroscope (Technica, Germany) by the same author, under identical magnification (×20) (Figure 1). The minimum distance from the canal wall of the MB1 and MB2 to the furcation area was measured, using a digital ruler in Adobe Photoshop 7.0. To standardize the measurements, a ruler was placed next to the tooth in the photographs and the measurements of the digital ruler were collaborated with this one. These values were then converted to millimeters. Two observers, working independently, measured all the dimensions. Where measurements difference was within 5% of each other, the average was calculated and recorded. However, thickness differences of greater than 5% were repeated by the observers during the same session until agreement was reached. The observers were blinded to the type of tooth they were examining (maxillary first or maxillary second molars).

### Data Analysis

All data were analyzed using STATA v.10. The mean FCWT was compared among different teeth by canal type (as between factors) and by location of sections (as within factor) while repeating the measurement Analysis of Variance. The analysis was completed by making paired comparisons using the Bonferroni alpha adjustment method. The general significance level was considered to exist if P<0.05 was met.

## RESULTS

**Figure 2:**
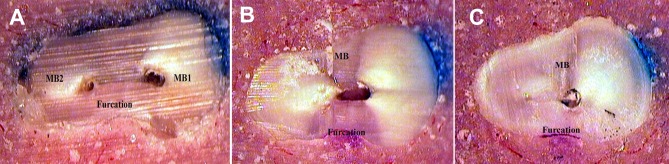
MB1 and MB2 canals of secondary maxillary molar. A) at CEJ, B) 2 mm lower CEJ, C) 4 mm lower CEJ. Note joining canals in 2 and 4 mm lower CEJ

**Figure 3:**
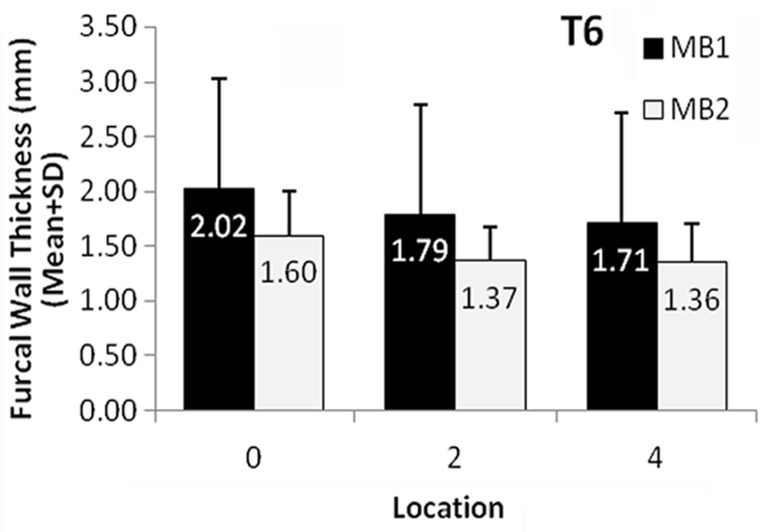
The mean Furcal Wall Thickness of MB1 and MB2 canal in three locations from CEJ in first maxillary molars (T6). Bars represent standard deviations. All the differences between MB1 and MB2 were statistically significant in all three locations

**Figure 4:**
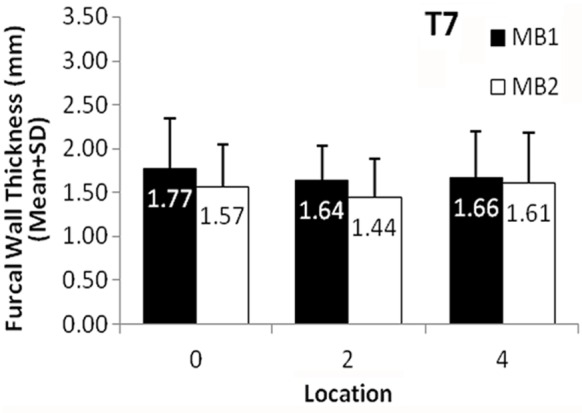
The mean Furcal Wall Thickness of MB1 and MB2 canal in three locations from CEJ in second maxillary molars (T7). Bars represent standard deviations. There was no significant differences between MB1 and MB2 in all three locations.

In this survey, 17 specimens were excluded from the study because their MB1 and MB2 canals joined (Figure 2). Only one of these specimens was a maxillary first molar, the rest were maxillary second molars. We also observed that four sections out of 17 were located 2 mm below the CEJ and 13 sections were located 4 mm below the CEJ. Therefore, 73 specimens remained for the analysis. The mean FCWT of MB1 and MB2 of the first and second maxillary molars at each level are shown in Figure 3 and Figure 4. The FCWT in the MB1 of the first maxillary molars at CEJ level was significantly greater than in MB2 (mean difference=0.42, CI 95%, 0.08-0.76, P<0.05). Moreover, the FCWT in the 2 mm and 4 mm segments below CEJ of the MB1 were also significantly greater than the MB2 (mean difference = 0.43, CI 95%, 0.16-0.69, P<0.05; mean difference = 0.35, CI 95% = 0.10-0.59, P<0.05, respectively).

In maxillary second molars, the FCWT in MB1 was greater than MB2 at CEJ; however this was not statistically significant (mean difference=0.20, CI95% -0.19 to 0.59, P>0.05). Also, the FCWT located 2 mm below CEJ in MB1 was not significantly greater than in MB2 (mean difference=0.2, CI 95%, -0.11 to 0.51). The FCWT located 4 mm below CEJ in MB1 was greater than in MB2; again however, this was not statistically significant (mean difference = 0.06, CI95% -0.36 to 0.47).

Overall, the FCWT of the second molars was greater in the MB1 than in MB2 at all levels, but the difference was not statistically significant.

## DISCUSSION

The FCWT in MB1 canals was greater than in MB2; especially in the maxillary first molars. Therefore, the clinician should be attentive when instrumenting the MB2 canals to avoid thinning of furcal canal walls [12]. It should be noted that in both MB1 and MB2 canals in the first maxillary molar, the dentine thickness decreased with increasing distance from CEJ. At 4 mm below the CEJ in MB2 canals, the mean FCWT was only 1.36 mm, increasing the risk of strip perforation in MB2 canals [12]. Anti-curvature filing must be considered to prevent strip perforation [13], and caution should be taken so as not to over-prepare the canal. Therefore, clinicians should be more meticulous when preparing the MB2 canal, having different requirements to the MB1 canal. Ni-Ti rotary instrumentation maintains the natural integrity and shape of the canal and reduces the risk of canal transportation and perforation than hand instrumentation techniques. Therefore, using nickel-titanium (Ni-Ti) rotary instruments is recommended in maxillary molars and in teeth with curved canals [14][15]. The initial thickness of the root canal dictates to what extent the instrumentation should be carried out to achieve thorough debridement and cleanliness. Other factors such as dentine thickness and the degree of the curvature of the canal are important risk factors of potential canal perforation. Also, these may lead to transporta-tion in the canal and over-preparation which can then increase the risk of perforation.

A total of 17 specimens were excluded from this study due to joining of MB1 and MB2, 16 of which were maxillary second molars, reducing the overall specimens in this group. Thus a lower statistical power of detecting the significant difference between MB1 and MB2 dentine thickness was obtained. When canals join, data could not be entered in either the MB1 group or the MB2 group. Other studies have sections up to 6-7 mm below CEJ [16][17]; however in this study, sectioning was performed to 4 mm as the authors believe that strip perforation is more probable. Moreover, there was no available research regarding dentine thickness of MB1 and MB2 canals of maxillary molars.

## CONCLUSION

In conclusion, in maxillary first molars, the FCWT in MB2 canals was less than in MB1 canals. However, there was no significant difference in FCWT in maxillary second molars. Further studies which investigate the remaining dentin canal wall thickness after instrument-tation are recommended.
